# Euonymus alatus in diabetes: a review of phytochemistry, pharmacokinetics, and anti-diabetic mechanisms

**DOI:** 10.3389/fphar.2026.1848743

**Published:** 2026-07-23

**Authors:** Li-Dan Xu, Chong-Yang Ma, Zhi-Yao Zhu, Tian An

**Affiliations:** 1 Capital Medical University, Beijing, China; 2 School of Traditional Chinese Medicine, Capital Medical University, Beijing, China; 3 Beijing Key Laboratory of TCM Collateral Disease Theory Research, Beijing, China

**Keywords:** diabetes mellitus, Euonymus alatus, mechanisms of action, pharmacokinetics, phytochemistry

## Abstract

Euonymus alatus: (EA), a traditional Chinese botanical drug documented in the Shennong Ben Cao Jing, has been investigated for its potential anti-diabetic effects. This review systematically examines the phytochemistry, pharmacokinetics, and anti-diabetic mechanisms of this botanical drug. Over 230 metabolites, including flavonoids, triterpenoids, and lignans, have been identified from EA. Pharmacokinetic studies remain limited; computational predictions suggest that some metabolites may exhibit oral bioavailability, but classical pharmacokinetic parameters have not been experimentally determined for any EA metabolite. Mechanistic studies demonstrate that EA exerts anti-diabetic effects through multiple experimentally validated pathways: (i) inhibiting alpha-glucosidase activity to delay intestinal glucose absorption; (ii) activating the peroxisome proliferator-activated receptor gamma and phosphatidylinositol 3-kinase/protein kinase B signaling pathways to ameliorate insulin resistance; (iii) modulating gut microbiota composition and increasing short-chain fatty acid production; (iv) suppressing the advanced glycation end products-receptor for advanced glycation end products axis along with the nuclear factor kappa B and mitogen-activated protein kinase inflammatory pathways to alleviate oxidative stress and inflammatory responses; and (v) regulating diacylglycerol acyltransferase activity to improve lipid metabolism. Preclinical studies indicate that EA reduces blood glucose and improves markers of diabetic nephropathy and retinopathy. Clinical studies of EA-containing formulations report reductions in fasting blood glucose and urinary protein. However, the clinical evidence remains limited by small sample sizes, lack of rigorous controls, and multi-botanical drug compositions that preclude attribution of effects to individual components. This review provides a critical synthesis of current evidence and identifies priorities for future investigation.

## 1 Introduction

Diabetes mellitus (DM) is a group of metabolic disorders characterized by chronic hyperglycemia, which has evolved into a global public health concern. According to the latest release of the *IDF Diabetes Atlas* published by the International Diabetes Federation, an estimated 589 million adults (aged 2–79 years) were living with DM worldwide in 2024, with approximately 90% of these cases corresponding to type 2 diabetes mellitus (T2DM) (International Diabetes Federation, 2025). The rising prevalence is primarily driven by the obesity epidemic, population aging, unhealthy dietary patterns, and sedentary lifestyles (NCD Risk Factor Collaboration NCD-RisC, 2024). Prolonged hyperglycemia can induce multi-organ complications, such as cardiovascular disease, kidney disease, retinopathy, and neuropathy. DM and its complications cause approximately 3.4 million deaths annually worldwide, imposing a heavy burden on patients’ quality of life and healthcare resources. Although currently available anti-diabetic drugs can control blood glucose, some of them are associated with side effects such as hypoglycemia, weight gain, and gastrointestinal reactions, and they often fail to achieve multi-target regulation (Gieroba et al., 2025). Therefore, the search for natural drugs with high efficacy, low-toxicity, and multi-mechanism actions has emerged as a key direction in T2DM research.

**FIGURE 1:**
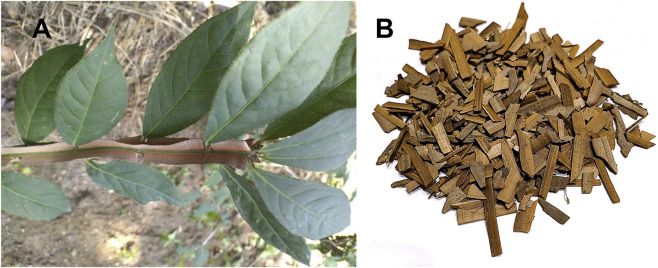
Representative images of *Euonymus alatus* (Thunb.) Siebold **(A)** and *Euonymi Alati Ramulus* **(B)**, which were used for isolating metabolites and treating DM.

*Euonymus alatus* (Thunb.) Siebold (EA, Figure 1), known as Guijianyu in Chinese, is the dried winged branch of *E. alatus*, a plant belonging to the family Celastraceae, genus *Euonymus*. EA was first recorded in the *Shennong Ben Cao Jing* (Shennong’s Classic of Materia Medica) and officially documented in *the Pharmacopoeia of the People’s Republic of China*, whose official pharmacopoeia name is Euonymi Alati Ramulus (Guijianyu). According to traditional Chinese medicine theories, it acts to break and dispel blood stasis, unblock the meridians and expel parasites (Zhao et al., 2010). Modern pharmacological studies have reported that EA extracts and their active metabolites exhibit multiple biological activities, including hypoglycemic, lipid-regulating, anti-inflammatory, and antioxidant effects, which warrants further investigation into their potential value in the prevention and treatment of T2DM and its complications (Xing et al., 2005). In recent years, the anti-T2DM mechanisms of EA have been increasingly investigated, involving amelioration of insulin resistance, protection of pancreatic *β* (beta)*-*cell function, regulation of gut microbiota, and inhibition of inflammatory responses. This article systematically synthesizes the relevant literature on EA and reviews from three perspectives: phytochemical basis, pharmacokinetic properties, and anti-T2DM mechanisms, with the aim of offering data-driven support for further research and clinical translation of EA.

## 2 Methodology

**FIGURE 2:**
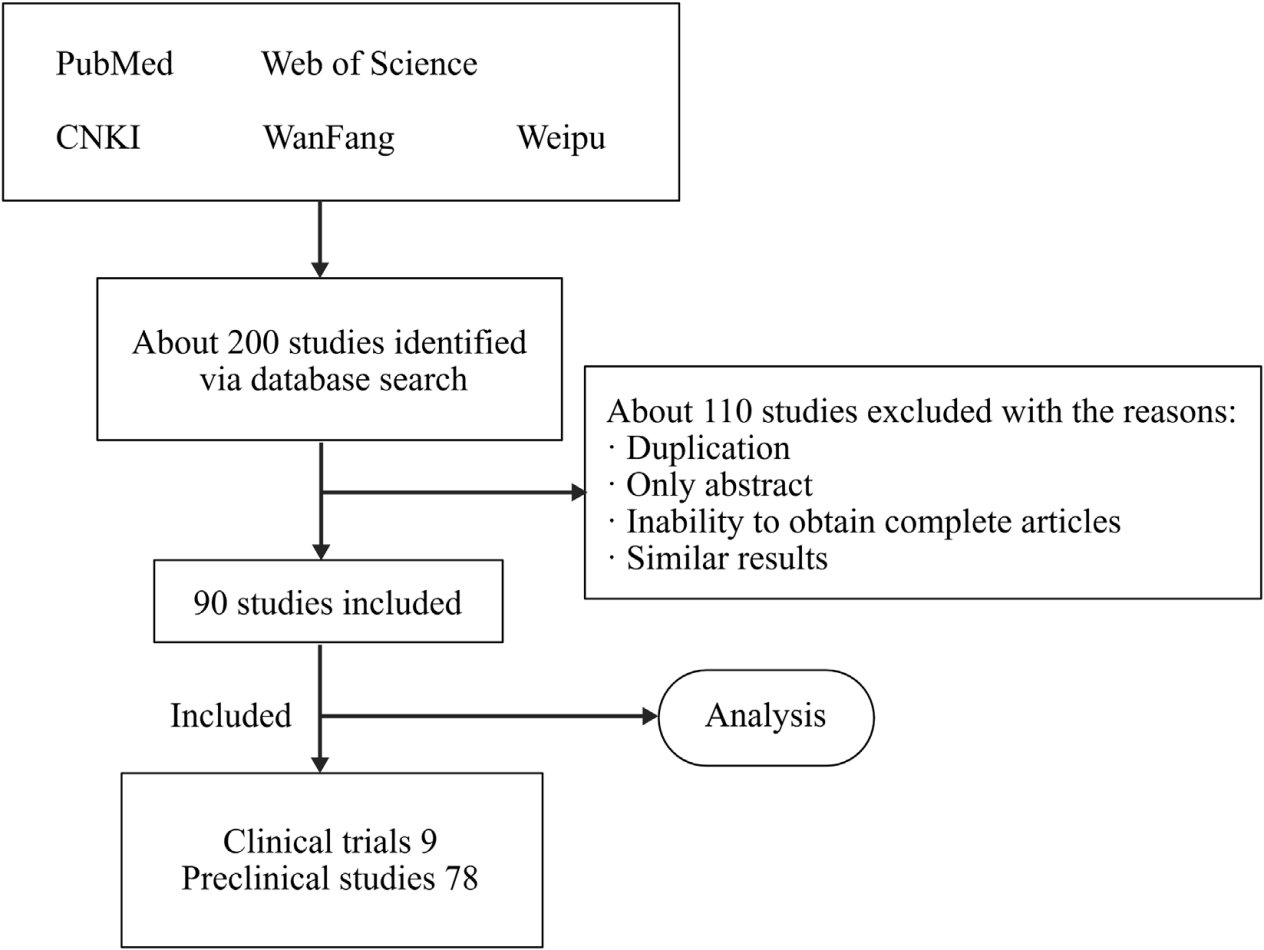
Flow chart of the literature analysis.

A systematic literature search was conducted to identify relevant studies on the anti-diabetic effects of *Euonymus alatus* (Thunb.) Siebold (*Euonymi Alati Ramulus*). The search was performed using the following electronic databases: PubMed (www.pubmed.com), China National Knowledge Infrastructure (www.cnki.net), Web of Science (www.webofscience.com), Weipu (www.cqvip.com), and Wanfang Data (www.wanfangdata.com). A detailed flowchart of the literature screening process is presented in Figure 2.

The following search terms were applied for literature retrieval: “*Euonymus alatus*”, “Guijianyu”, “diabetes mellitus”, “hyperglycemia”, “insulin resistance”, “anti-diabetic”, “hypoglycemic”, “type 2 diabetes”, “glucose metabolism”, “insulin signaling”, as well as the names of major phytochemicals such as flavonoids, terpenoids, and coumarins. Both Chinese and English keywords were used according to the language of each database. The titles and abstracts of all retrieved records were screened for eligibility. While the standardized scientific name *E. alatus* and its common Chinese name were used to ensure comprehensiveness, a small number of very early publications employing historical taxonomic synonyms (e.g., Celastrus genus nomenclature) may not have been fully retrieved. To address this, the search terms included both *E. alatus* and *Celastrus* to capture publications using historical binomial nomenclature. However, this limitation does not influence the main conclusions of this comprehensive review.

A total of approximately 200 papers were retrieved from database inception to March 2026. Among these, around 110 papers were excluded based on the following criteria: duplicate records, conference abstracts without full-text availability, and studies with redundant or overlapping results. The remaining 90 papers were included for further analysis, with a focus on phytochemistry, pharmacokinetics, pharmacological mechanisms, and clinical applications related to the anti-diabetic effects of this botanical drug. The extracted data were systematically summarized and categorized to provide a comprehensive overview of the current research on EA for diabetes management.

To objectively evaluate the reliability of retrieved data and unify the presentation logic of this review, a three-tier evidence grading system is applied to all included studies across phytochemistry, pharmacokinetics, anti-diabetic mechanisms and clinical research. The grading criteria are defined as follows:

Strong evidence: supported by consistent *in vitro*, animal, and clinical data.

Moderate evidence: supported by robust *in vitro* and animal data, with limited clinical evidence.

Preliminary evidence: supported primarily by *in vitro* studies.

## 3 Phytochemical research

The chemical composition of EA is complex, with over 230 metabolites isolated and identified to date. In this review, EA refers to the dried winged twigs of *E. alatus* (Thunb.) Siebold (Guijianyu), and most studies involve extracts prepared with ethanol, water, or organic solvents via reflux, ultrasonic or microwave-assisted extraction methods. Its main active metabolites include flavonoids, terpenoids, phenylpropanoids, lignans, and alkaloids (Acharya et al., 2024; Fan et al., 2020). Most studies use hyphenated techniques of chromatography and spectroscopy for separation and identification.

### 3.1 Extraction and separation methods (preliminary evidence)

(Lee et al., 2024) found that the anti-DM effect of EA’s ethanol extract was significantly superior to that of its aqueous extract, mainly because ethanol extracts a greater amount of flavonoids. Ba et al. (2012) adopted reflux extraction with 95% ethanol to process EA, followed by separation via silica gel column chromatography and Sephadex LH-20 gel column chromatography, and identified nine metabolites (including apigenin and linarin) from the ethyl acetate fraction. Fang (2007), Jung et al. (2020) used similar methods and separately isolated 19 metabolites, ten of which were new natural products, such as 7-methoxy-4-methylisobenzofuranone and betulin. Cha et al. (2003), Seo et al. (2025) discovered that EA’s butanol extract exhibited strong inhibitory activity against matrix metalloproteinase-9 (MMP-9) (with a half-maximal inhibitory concentration (IC_50_) as low as 10 μg/mL), and its inhibitory effect surpassed that of chloroform or hexane extracts. Microwave-assisted extraction technology promotes metabolite release by disrupting cell walls, reducing both solvent consumption and extraction time. Yang and Zhang (2008) used an ultrasonic-assisted extraction method to extract rutin and quercetin from dried EA stems. Zhang et al. (2009) reported that extraction with 50% ethanol at a microwave power of 170 W for 6 min efficiently yielded rutin and quercetin, with a higher extraction rate than Soxhlet extraction and ultrasonic extraction. Mai et al. (2020) employed a combination of ultrasonic-assisted extraction and temperature-induced cloud point extraction to successfully separate and identify metabolites from EA, including catechin, dihydromyricetin, gallic acid, and ellagic acid.

### 3.2 Metabolite identification (preliminary evidence)

Chen et al. (2010) established a high-performance liquid chromatography method to determine the content of aurantiamarin in EA, using a Kromasil C18 column with acetonitrile-0.3% acetic acid as the mobile phase and a detection wavelength of 292 nm. Methodological validation showed good linearity (r = 0.9999). Yoon et al. (2024) analyzed the ethanolic leaf extract via high-performance liquid chromatography and identified chlorogenic acid and lucidin glycoside as the main antioxidant metabolites, with contents of 2.07 mg/g and 11.09 mg/g, respectively. Li et al. (2021), Hao et al. (2017) identified various saponins (e.g., ginsenosides Rb1 and Rg1) and lignans via liquid chromatography-mass spectrometry, which are closely related to the pharmacological activities of EA. Wang et al. (2025) identified 56 metabolites (e.g., palmitic acid and wintergreen oil) from the volatile oil of EA using gas chromatography-mass spectrometry. Li et al. (2026) identified eight flavonoid metabolites in EA via ultra-performance liquid chromatography–quadrupole time-of-flight mass spectrometry. Yamashita et al. (2019) isolated four new triterpenes from the bark and determined their structures via nuclear magnetic resonance and X-ray diffraction, revealing that lupane-type triterpenes are the core active metabolites of EA. Park W H. et al. (2005), Jin et al. (2005) identified caffeic acid and chlorogenic acid as the main active metabolites via high-performance liquid chromatography and mass spectrometry. The structure of caffeic acid was confirmed by fast atom bombardment mass spectrometry and nuclear magnetic resonance, with a molecular formula of C_9_H_8_O_4._ The caffeic acid inhibited MMP-9 enzyme activity in a concentration-dependent manner (IC_50_ of 10–20 nM). Xiao (2013) identified novel metabolites including catechin lactone A from EA.

### 3.3 Quantitative analysis (preliminary evidence)

Dang et al. (2014) compared the NaNO_2_-Al(NO_3_)_3_-NaOH method, AlCl_3_ method, and HCl-Mg method for determining total flavonoid content, finding that the HCl-Mg method had high precision (relative standard deviation = 2.220%) and was suitable for EA quality control. Zhang (2007) optimized the extraction process of rutin and quercetin from EA using microwave-assisted extraction–high performance liquid chromatography. The optimal conditions were 60% ethanol, a liquid-to-solid ratio of 40:1, a microwave power of 255 W, and extraction for 6 min, yielding an extraction efficiency comparable to Soxhlet extraction but requiring less time. Zhai et al. (2016), Yoon et al. (2024) quantitatively determined metabolites such as quercetin, kaempferol, and their glycosidic derivatives (e.g., isoquercitrin and rutin) using ultra-performance liquid chromatography-tandem mass spectrometry, and found that these metabolites were more abundant in leaves and seed wings.

## 4 Pharmacokinetic properties

In silico screening tools such as the Traditional Chinese Medicine Systems Pharmacology Database have been used to computationally predict the oral bioavailability of EA metabolites. However, such predictions use arbitrary threshold criteria (e.g., OB ≥ 30%) that lack experimental validation and do not account for species-specific differences in intestinal permeability, hepatic first-pass metabolism, or the complex food matrix effects associated with botanical drug extracts. These computational estimates should be regarded as hypothesis-generating rather than established pharmacokinetic facts. Standard *in vivo* pharmacokinetic parameters including plasma concentration-time curves, maximum concentration, area under the curve, and half-life have not been determined; therefore, direct evidence for therapeutically relevant plasma concentrations in humans is currently insufficient.

### 4.1 Prediction of metabolite absorption and distribution (preliminary evidence)

Woo et al. (2020) found that rutin is hydrolyzed into quercetin by gut microbiota. The resulting quercetin can be directly absorbed via the small and large intestines and can maintain a relatively high plasma concentration, thereby enhancing its antioxidant and anti-inflammatory activities. Jeong et al. (2011a) found that the activities of quercetin-3-O-*β*-D-galactoside and apigenin-3-O-*α* (alpha)-L-rhamnoside are weaker compared with their aglycones (quercetin and apigenin), which may be related to glycosylation affecting their cell membrane permeability, indicating that glycosylation of flavonoids may affect their absorption and biological activity. Ji et al. (2024) found that the steroidal metabolite stigmast-4-en-3-one has an OB of 36.08% and drug-likeness of 0.76, conforming to Lipinski’s Rule of Five. Lee et al. (2024) found that after administration, the extract distributed to the liver and muscle tissues and activated the insulin signaling pathway, indicating that it can cross biological barriers to reach target organs. Kim et al. (2024) found that protocatechuic acid, as one of the metabolites of EA, can cross the blood-brain barrier, maintain high plasma concentrations, and exert antioxidant effects. Gurung et al. (2023) showed that quercetin in EA extract can cross the blood-brain barrier, activate the extracellular signal-regulated kinase, cyclic adenosine monophosphate response element-binding protein and brain-derived neurotrophic factor pathway, and improve cognitive function. Kim et al. (2024) also found that quercetin at a dose of 10 mg/kg significantly ameliorated memory impairment in mice, which was related to its high bioavailability.

It should be noted that quercetin, kaempferol, and rutin, frequently reported as putative active metabolites of EA, are classified as Pan-Assay Interference Compounds known to produce artifactual positive results in various *in vitro* assays through non-specific mechanisms including aggregation, redox cycling, and protein reactivity (Bolz et al., 2021). *In vitro* activity data for these compounds must therefore be interpreted with caution and confirmed using Pan-Assay Interference Compounds-filtered assays before they can be considered validated pharmacological agents.

### 4.2 Pharmacokinetic experimental studies (preliminary evidence)

Li et al. (2024) pointed out that the combination of EA and *Astragalus mongholicus* Bunge (Fabaceae; official pharmacopoeia name: Astragali Radix (Huangqi)) might affect metabolite distribution via the peroxisome proliferator-activated receptor gamma (PPAR*γ*) pathway, but this finding lacks validation using *in vivo* pharmacokinetic parameters. Wang (2022) conducted rat experiments and found that EA could improve vascular endothelial function, reduce blood pressure, and downregulate vascular endothelial injury markers (endothelin-1 and von Willebrand factor) by inhibiting endoplasmic reticulum stress mediated by the protein kinase R-like endoplasmic reticulum kinase pathway. This suggests that its metabolites may distribute to vascular tissues after intestinal absorption. Zhang et al. (2017) showed that the Man-Pen-Fang metabolite prescription (containing EA), when orally administered to rat models, exerted anti-inflammatory activities, implying that its metabolites might be absorbed through the gastrointestinal tract and distributed to target tissues. Zhang (2018) reported that EA reduced urinary protein excretion in DM patients, which indirectly reflects the renal distribution characteristics of its metabolites. Lee et al. (2024) concluded from cell experiments that EA extract at 50 μg/mL resulted in less than 50% cytotoxicity against rat insulinoma INS-1 cells, suggesting high safety for short-term use. However, existing studies have not investigated classic pharmacokinetic parameters such as plasma concentration-time curves and half-life, indicating that further pharmacokinetic investigations are needed.

### 4.3 Metabolic pathways (preliminary evidence)

Glucuronidation represents the primary phase II metabolic pathway for flavonoid metabolites in mammals. Quercetin, a major flavonoid in EA, undergoes extensive hepatic glucuronidation following absorption, with quercetin-3-O-glucuronide and quercetin-7-O-glucuronide as principal metabolites. These transformations substantially alter the physicochemical properties and biological activity of the parent compound. Computational predictions of metabolic enzyme interactions (e.g., cytochrome P450 binding (Bu et al., 2021) have been reported but remain to be validated experimentally. Zhou et al. (2021) investigated the potential interaction of aromadendrin with PPARγ in vascular endothelial cells; however, the study did not report classical pharmacokinetic parameters. Ji et al. (2024) carried out *in vivo* rat experiments and confirmed that stigmast-4-en-3-one exhibited good affinity for ocular tissues, indicating its ability to cross the blood-retinal barrier and exert therapeutic effects. It also has good lipophilicity, is easily absorbed in the intestine, undergoes normal metabolism and excretion, and displays no mutagenicity with low toxicity.

## 5 Anti-DM mechanisms of action

For anti-DM mechanisms, each individual molecular pathway and biological activity is graded independently (same as above standard) according to its own available data.

### 5.1 *In Vitro* experimental studies

This section presents preliminary *in vitro* evidence characterizing biological activities and molecular targets.

#### 5.1.1 *α*-Glucosidase inhibitory activity (moderate evidence)

Lee et al. (2007) employed a yeast *α*-glucosidase assay with p-nitrophenyl-*α*-D-glucopyranoside as the substrate, determining an IC_50_ value of 0.272 mg/mL, which was superior to acarbose (IC_50_ > 0.5 mg/mL). In a streptozotocin (STZ)-induced DM rat model, oral administration of EA extract after starch loading significantly reduced the postprandial blood glucose peak (p < 0.05 at 60 and 90 min) and decreased the area under the blood glucose curve. The mechanism involves direct inhibition of intestinal *α*-glucosidase, delaying carbohydrate hydrolysis and thereby controlling postprandial hyperglycemia.

#### 5.1.2 Regulation of insulin signaling pathways (moderate evidence)

PPAR*γ* Pathway: Yang et al. (2004) established an insulin resistance model in rat adipocytes induced by high glucose and insulin, and found that EA extract promoted glucose uptake. This effect was associated with the activation of PPAR*γ*. Park S. H. et al. (2005) reported that EA ethanol extract upregulated PPAR*γ* mRNA expression in mature epididymal adipose tissue of mice. Fang et al. (2008) demonstrated that kaempferol and quercetin isolated from EA acted as weak partial agonists of PPAR*γ*. Unlike conventional full PPAR*γ* agonists (e.g., rosiglitazone), kaempferol and quercetin exhibited an “uncoupling” effect. They markedly elevated glucose uptake in insulin-stimulated mature 3T3-L1 adipocytes, without activating or antagonizing the gene transcription programs associated with adipogenesis.

**FIGURE 3:**
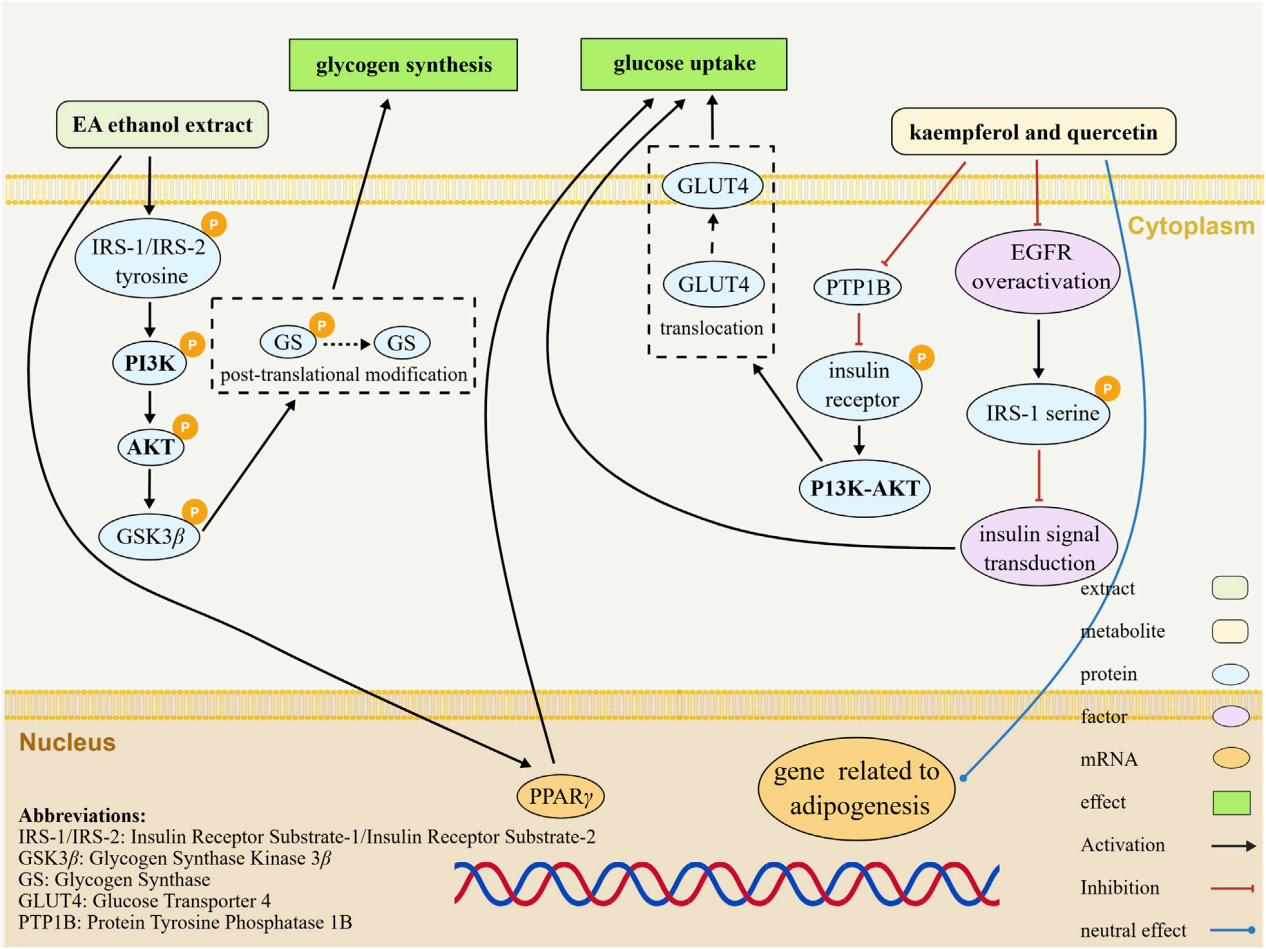
Schematic Diagram Illustrating the Anti-diabetic Mechanism of EA via Regulation of insulin signaling pathway.

Phosphatidylinositol 3-kinase (PI3K) and protein kinase B (AKT) Pathway: Fan et al. (2020); Jeong et al. (2015) found that quercetin (IC_50_ = 5.43 μM) and kaempferol (IC_50_ = 4.97 μM) derived from EA competitively inhibited protein tyrosine phosphatase 1 B. These compounds enhanced insulin receptor phosphorylation, activated the PI3K/AKT pathway, and facilitated glucose transporter four translocation, thereby increasing cellular glucose uptake. Acharya et al. (2024), Lee et al. (2024) observed that the 70% ethanol extract of EA promoted tyrosine phosphorylation of insulin receptor substrate-1 and insulin receptor substrate-2, which further triggered the activation of PI3K. Activated PI3K induces the phosphorylation of AKT, which in turn phosphorylates glycogen synthase kinase 3*β* and renders it inactive. This process results in dephosphorylation of glycogen synthase, accelerating glycogen synthesis in the liver and skeletal muscle. Wang et al. (2024) indicated that quercetin and kaempferol improved insulin signal transduction by inhibiting excessive activation of the epidermal growth factor receptor (EGFR) and reducing serine phosphorylation of insulin receptor substrate-1, thus exerting a synergistic hypoglycemic effect. Anti-diabetic mechanism of EA via regulation of insulin signaling pathway is illustrated in the Figure 3 below.

#### 5.1.3 Gut microbiota regulation mechanism (moderate evidence)

He et al. (2022) demonstrated that the combination of EA and *Potentilla discolor* Bunge (Rosaceae; official pharmacopoeia name: Potentillae Discoloris Herba (Fanbaicao)) significantly improved gut microbiota dysbiosis in T2DM rats, reducing the Firmicutes/Bacteroidetes ratio (from 3.40 to 1.34) and increasing the abundance of *Lactobacillus* and *Akkermansia*. The mechanism was related to elevated production of short-chain fatty acids (e.g., butyrate), which improved insulin sensitivity by activating G-protein-coupled receptor 43.

#### 5.1.4 Oxidative stress and apoptosis mechanisms (moderate evidence)

Li et al. (2026) reported that kaempferol and polyphenolic metabolites in EA could reduce the expression of NADPH oxidase four and alleviate damage to renal tubular epithelial cells. Wang et al. (2021) found that the flavonoid metabolite rhamnazin-3-O-rutinoside inhibited oxidative stress responses by scavenging free radicals. Sun et al. (2025) found that total polyphenols and flavonoids protected *β*-cell function by reducing oxidative damage and inhibiting apoptotic proteins. Kim et al. (2024), Shin et al. (2023) found that protocatechuic acid and quercetin could activate the nuclear factor erythroid 2-related factor 2 pathway, upregulate the expression of heme oxygenase-1, superoxide dismutase, and glutathione peroxidase, reduce reactive oxygen species levels, and alleviate oxidative stress-induced damage to *β* cells. Wu (2021) induced renal tubular epithelial cell damage with doxorubicin and confirmed that EA could reduce reactive oxygen species production and malondialdehyde levels, and increase superoxide dismutase activity, thereby alleviating oxidative stress.

**FIGURE 4:**
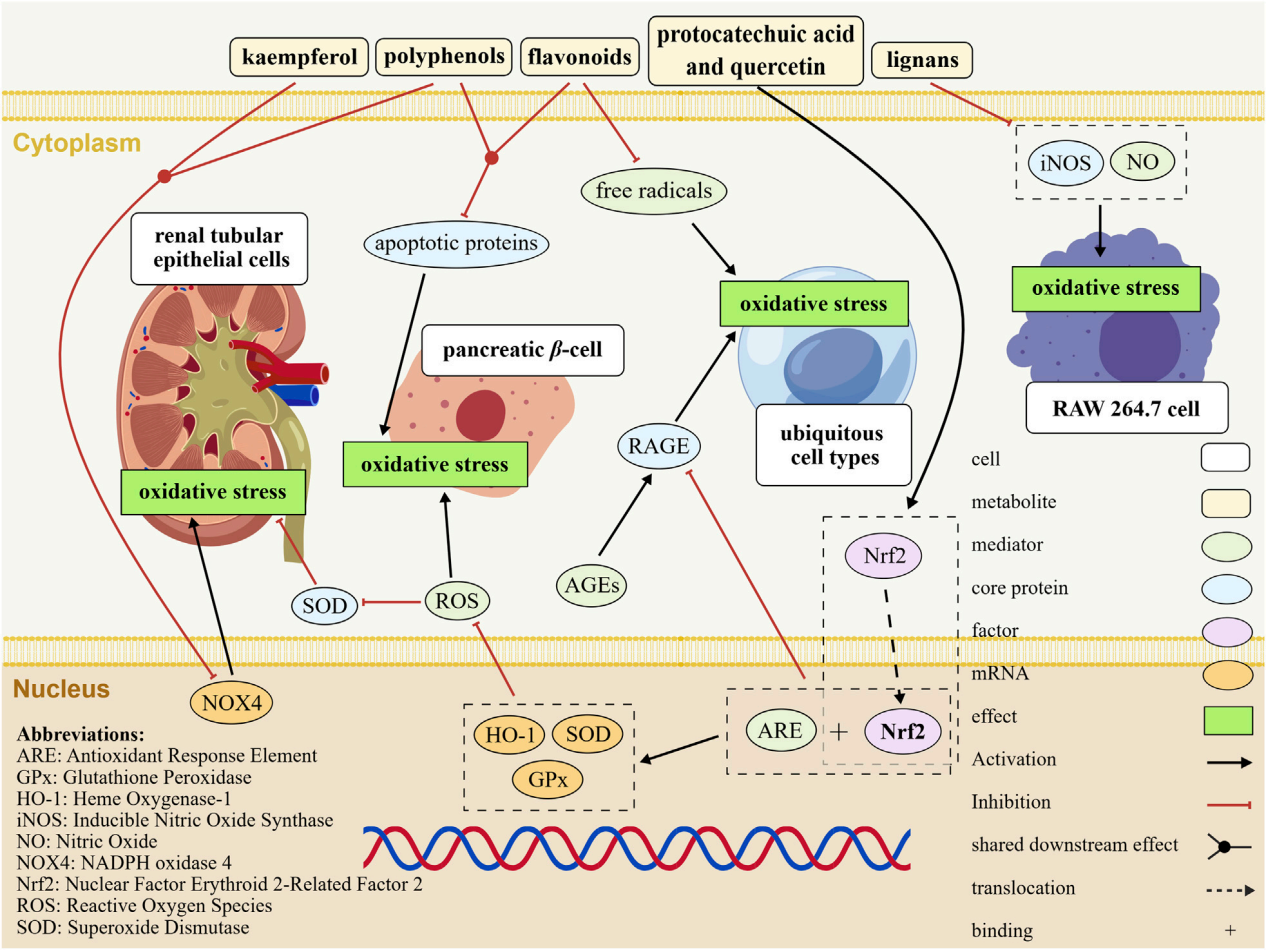
Schematic Diagram Illustrating the Anti-diabetic Mechanism of EA through Modulation oxidative stress.

Chen et al. (2022) found that EA cleared reactive oxygen species via the nuclear factor erythroid 2-related factor 2 and antioxidant response element pathway and inhibited the advanced glycation end products (AGEs)-receptor for advanced glycation end products (RAGE) axis. By suppressing RAGE expression, it reduced receptor activity and blocked AGEs-induced oxidative stress. Oh et al. (2011), Jeong et al. (2011b) found that in RAW 264.7 cells, lignan metabolites from EA could inhibit lipopolysaccharide (LPS)-induced inducible nitric oxide synthase expression and excessive nitric oxide production (IC_50_ values of 8.5–12.8 μM), thereby alleviating oxidative stress. Anti-diabetic mechanism of EA through modulation oxidative stress is illustrated in the Figure 4 below.

#### 5.1.5 Regulation of inflammatory pathways (moderate evidence)

Huang et al. (2014) found that the water extract of EA could inhibit the release of tumor necrosis factor-*α* (TNF-α) and interleukin-6 (IL-6) from human monocytic macrophages induced by oxidized low-density lipoprotein. Wan et al. (2019) found that the ethanol extract of EA inhibited TNF-*α*-induced nuclear factor-kappa B (NF-*κ*B) activation, thereby alleviating inflammatory response. Lee et al. (2019) found that (3*β*,16*α*)-3,16-dihydroxypregn-5-en-20-one significantly reduced the expression of inducible nitric oxide synthase and cyclooxygenase-2 by inhibiting LPS-induced activation of the NF-*κ*B and mitogen-activated protein kinase and extracellular signal-regulated kinase signaling pathways. Oh et al. (2011) pointed out that EA extract can block the NF-*κ*B inflammatory pathway by inhibiting I*κ*B kinase *β*, and (Acosta-Martinez and Cabail, 2022; Liu et al., 2014) noted that crosstalk existed between the I*κ*B kinase *β* and NF-*κ*B pathway, PI3K/AKT pathway, and mitogen-activated protein kinase and extracellular signal-regulated kinase pathway, suggesting that inhibiting inflammation may improve insulin signal transduction.

**FIGURE 5:**
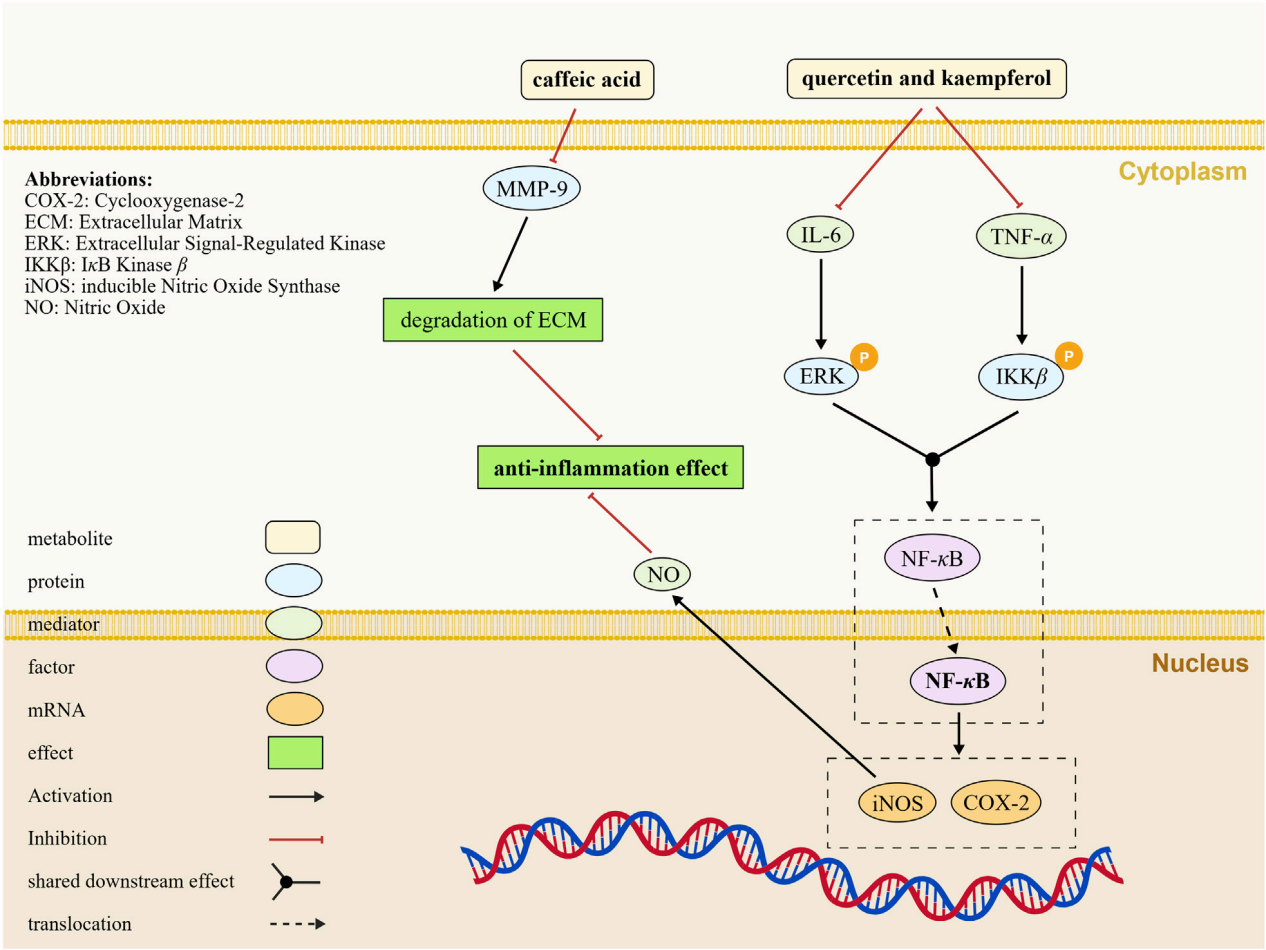
EA exerting anti-diabetic effects through modulation of inflammatory pathways.

Seo et al. (2005) found that the butanol extract of EA strongly inhibited the gelatinolytic activity of MMP-9. Park W. H. et al. (2005) further isolated caffeic acid from EA and confirmed that it directly inhibited the catalytic activity of MMP-9, thereby preventing degradation of the extracellular matrix and exerting anti-inflammatory activities. Anti-diabetic mechanism of EA through modulation of inflammatory pathways is illustrated in the Figure 5 below.

#### 5.1.6 Immune regulation mechanism (moderate evidence)

Chung et al. (2002) found that the EA extract synergized with interferon-*γ* to increase the production of nitric oxide and TNF-α, while an NF-*κ*B inhibitor could block this effect. This indicates that EA extract promoted the expression of inflammatory mediators via the NF-*κ*B signaling pathway, thereby exerting immune regulatory effects.

#### 5.1.7 Diacylglycerol acyltransferase (DGAT) enzyme regulation mechanism (preliminary evidence)

Bansal and Durrett (2016), Milcamps et al. (2005) found that EA seeds contain a special lipid accounting for up to 95% of their content — 1,2-long-chain-diacyl-3-acetyl-sn-glycerol. The key enzyme for synthesizing this acetylated triacylglycerol is acetyl-CoA acetyltransferase. Studies have shown that the diacylglycerol acyltransferase one gene cloned from developing EA seeds produces an enzyme with acetyl-CoA acetyltransferase activity, which preferentially catalyzes the incorporation of acetyl groups into the *sn*-3 position of triacylglycerols (TAGs), indicating that this is a DGAT enzyme with unique substrate promiscuity. Durrett et al. (2010) confirmed that *Euonymus alatus* diacylglycerol acetyltransferase (EaDAcT) was a specialized DGAT and the primary enzyme for 1,2-long-chain-diacyl-3-acetyl-sn-glycerol synthesis. 1,2-long-chain-diacyl-3-acetyl-sn-glycerol have a viscosity 30% lower than that of common TAGs, which may reduce cellular lipid deposition and alleviate lipotoxicity-induced interference with insulin signaling. This aligns with the observation by Park S. H. et al. (2005) that liver TAGs content in mice treated with EA extract decreased by 72%–77%, indirectly improving the insulin signaling pathway, suggesting the pathway of EaDAcT’s anti-DM effect. However, the lack of *in vivo* DM models prevents direct evidence of EaDAcT’s regulation of blood glucose, and extract studies cannot rule out contributions from other metabolites. Future studies are required to purify EaDAcT and conduct DM model experiments to verify causality.

### 5.2 Animal experimental studies

#### 5.2.1 Regulation of glucose and lipid metabolism (moderate evidence)

Chen et al. (2018) used an STZ-induced DM mouse model and treated with EA alone or in combination with botanical drugs such as *Gynostemma pentaphyllum* (Thunb.) Makino (Cucurbitaceae; official pharmacopoeial name: Gynostemmae Herba (Jiaogulan)). The treatment dose-dependently reduced fasting blood glucose and improved glucose tolerance, with the high-dose group (200 mg/kg) showing particularly significant effects—blood glucose returned to normal levels within 3 weeks. Li J. E. et al. (2010) prepared a model by feeding a high-fat diet combined with low-dose STZ injection, dividing animals into a model group, EA high/low-dose groups, and a pioglitazone control group. After 4 weeks of gavage treatment, the EA high-dose group (6 g/kg), significantly decreased fasting blood glucose and fasting insulin levels, increased the insulin sensitivity index, reduced levels of TAGs, total cholesterol, and low-density lipoprotein, and elevated high-density lipoprotein levels. Duan and Wu (2014) also demonstrated in a *Drosophila* model that co-administration of EA and *Litchi chinensis* Sonn. (Sapindaceae; official pharmacopoeial name: Litchi Semen (Lizhihu)) attenuated high-glucose-induced or high-fat-induced elevation of trehalose and TAGs, suggesting a conserved role across species in glucose and lipid regulation.

#### 5.2.2 Target organ protection (moderate evidence)

Zhao et al. (2010) reported that in a T2DM rat model, EA protected the morphology of pancreatic *β* cells, increased the area of insulin-positive staining, reduced blood glucose. Further immunohistochemical analysis confirmed it promoted the functional recovery of *β* cells. Chen et al. (2022) found in a rabbit renal ischemia-reperfusion injury model that the ethanol extract of EA inhibited the transforming growth factor-*β* receptor 1-SMAD family member 2/3 protein pathway, alleviating renal tissue fibrosis. Zhang et al. (2011) used a rat diabetic nephropathy (DN) model prepared by STZ injection combined with unilateral nephrectomy. After 12 weeks of gavage treatment with an EA-containing metabolite preparation (containing EA, *Astragalus mongholicus*, *Salvia miltiorrhiza* Bunge (Lamiaceae; official pharmacopoeial name: Salviae Miltiorrhizae Radix et Rhizoma (Danshen)), *etc.*), the expression of SMAD family member four protein in renal tissue was downregulated, and SMAD family member seven protein expression was upregulated, thereby inhibiting glomerulosclerosis mediated by transforming growth factor-*β*1. Pathological examination showed that mesangial proliferation and collagen deposition in the glomeruli of the treatment group were reduced, and Masson staining indicated improved fibrosis. Ma et al. (2025) found in a db/db spontaneous DM mouse model that the medium-dose and high-dose groups of EA (1.08 g/kg, 2.16 g/kg) could reduce serum creatinine, blood urea nitrogen, and urinary protein, alleviate renal pathological damage (such as basement membrane thickening and foot process fusion), and downregulate the expression of key proteins in the AGEs-RAGE signaling pathway (e.g., RAGE, NF-*κ*B, IL-6), indicating that it delayed the progression of DN through anti-inflammatory and anti-oxidative stress mechanisms.

#### 5.2.3 Differences in effects of different extracted parts (moderate evidence)

(Qi et al., 1998) induced a DM model in mice via intraperitoneal injection of alloxan, divided EA into five extracted parts, and administered them by gavage at a raw herb dose of 10 g/kg for 14 days. All extracted parts of EA showed hypoglycemic effects, among which the water decoction part was the most potent, dose-dependently reducing fasting blood glucose and improving glucose tolerance. Li Y. J. et al. (2010) showed that after inducing a DM model in rats by intraperitoneal injection of STZ, the rats were divided into a model group and treatment groups with different extracted parts of EA. The results showed that the petroleum ether, water, and ethyl acetate fractions of EA significantly reduced blood glucose levels (P < 0.05), while the n-butanol and refined polysaccharide fractions were more effective in reducing blood creatinine and blood urea nitrogen. The residue fraction reduced low-density lipoprotein and cholesterol levels, the water fraction reduced blood viscosity, and the refined polysaccharide fraction increased hematocrit. These results indicated that EA acted through multi-target mechanisms, affecting different links in DM pathogenesis (such as hypoglycemic, lipid-regulating, and hemorheological improvement), thereby preventing and treating DM and its complications.

### 5.3 Clinical studies

Clinical observations represent the highest level of evidence. However, most studies are based on traditional Chinese medicine formulas that cannot be attributed to EA alone, and thus are classified as moderate evidence.

#### 5.3.1 Compatibility of traditional Chinese medicine formulas and their therapeutic effects (moderate evidence)

EA is one of the core drugs used by Professor Zhou Zhongying (Tian et al., 2014) in treating DM, with a usage frequency of 126 times. Professor Zhou (Li, 2024) combined EA with *Rehmannia glutinosa* (Gaertn.) Libosch. ex DC. (Orobanchaceae; official pharmacopoeia name: Rehmanniae Radix (Shengdi)) and *Paeonia × suffruticosa* Andrews (Paeoniaceae; official pharmacopoeia name: Moutan Cortex (Mudanpi)) to cool blood and resolve stasis, thereby improving microvascular complications of DM. Zhu (1999) used the Liver-Soothing and Qi-Regulating Formula (containing EA) to treat 100 patients, achieving a total effective rate of 91%. Professor Guo Junjie (Song, 2020) pointed out that EA “could improve insulin resistance and achieve remarkable hypoglycemic effects”, and is often used in combination with *Paeonia × suffruticosa* and *Curcuma aromatica* Salisb. (Zingiberaceae; official pharmacopoeia name: Curcumae Radix (Yujin)) to form a herbal pair, thereby enhancing the effect of removing stasis. The Astragalus-EA Formula (containing *A. mongholicus* and EA) could lower blood glucose, urinary protein, and inflammatory factors (e.g., TNF-α, IL-6), inhibit the expression of cysteine-aspartic acid protease three in renal tissue, thereby treating DN.

Liang and Zhang (2001) used Leis Aromatic Turbidity-Resolving Formula (EA 15g, *Agastache rugosa* (Fisch. and C.A.Mey.) Kuntze (Lamiaceae; official pharmacopoeia name: Pogostemonis Herba (Huoxiang)) 15g, *Eupatorium japonicum* Thunb. (Asteraceae; official pharmacopoeia name: Eupatorii Herba (Peilan)) 15g, *Citrus reticulata* Blanco (Rutaceae; official pharmacopoeia name: Citri Reticulatae Pericarpium (Chenpi)) 10g, *Pinellia ternata* (Thunb.) Makino (Araceae; official pharmacopoeia name: Pinelliae Rhizoma (Banxia)) 10g, *Magnolia officinalis* Rehder and E.H.Wilson (Magnoliaceae; official pharmacopoeia name: Magnoliae Officinalis Cortex (Houpo)) 10g, *Areca catechu* L. (Arecaceae; official pharmacopoeial name: Arecae Pericarpium (Dafupi)) 10g, *Nelumbo nucifera* Gaertn. (Nelumbonaceae; official pharmacopoeial name: Nelumbinis Folium (Heye) 10g, *Scutellaria baicalensis* Georgi (Lamiaceae; official pharmacopoeia name: Scutellariae Radix (Huangqin)) 15g, *S. miltiorrhiza* 30 g) in a 57-patient treatment group. Among them, 30 cases showed good outcomes, 18 cases showed moderate outcomes, and the total effective rate was 84.2%. The metformin group had a total effective rate of 81.3%, with no significant difference between the two groups. However, the advantage of Lei’s Aromatic Turbidity-Resolving Formula lies in that it does not affect sleep and shows significant improvement in fasting blood glucose. Liu and Leng (2018) used the Qiyu Tangmai Kang Formula (*A. mongholicus* 40g, EA 20g, *Scrophularia ningpoensis* Hemsl. (Scrophulariaceae; official pharmacopoeial name: Scrophulariae Radix (Xuanshen)) 10g, *Ophiopogon japonicus* (Thunb.) Ker Gawl. (Asparagaceae; official pharmacopoeia name: Ophiopogonis Radix (Maidong)) 10g, *Dendrobium nobile* Lindl. (Orchidaceae; official pharmacopoeia name: Dendrobii Caulis (Shihu)) 10g, *Coptis chinensis* Franch. (Ranunculaceae; official pharmacopoeia name: Coptidis Rhizoma (Huanglian)) 3g, *Crataegus monogyna* Jacq. (Rosaceae; official pharmacopoeia name: Crataegi Fructus (Shanzha)) 10g, *Angelica sinensis* (Oliv.) Diels (Apiaceae; official pharmacopoeia name: Angelicae Sinensis Radix (Danggui)) 10g, *Conioselinum anthriscoides* ‘Chuanxiong’ (Apiaceae; official pharmacopoeia name: Chuanxiong Rhizoma (Chuanxiong)) 10g, fresh *N. nucifera* three pieces, *Glycyrrhiza uralensis* Fisch. ex DC. (Fabaceae; official pharmacopoeia name: Glycyrrhizae Radix et Rhizoma (Gancao)) 6 g) to treat 56 patients with stage III–IV DN. After treatment, fasting blood glucose decreased from 9.56 ± 2.19 mmol/L to 6.68 ± 1.63 mmol/L, and 24-h urinary protein quantification decreased from 2.56 ± 0.53 g/24h to 0.33 ± 0.73 g/24 h.

#### 5.3.2 Treatment of DM complications (moderate evidence)

DN Management: Qi et al. (2000) developed a metabolite EA preparation to treat chronic nephritis, achieving a total effective rate of 91.7%. Su (2011)’s data mining revealed that Professor Zhou Zhongying frequently combined EA with *E. japonicum* and *Lycium barbarum* L. (Solanaceae; official pharmacopoeia name: Cortex Lycii (Digupi)) in treating DN, which could lower blood glucose, regulate blood lipids, and slow arteriosclerosis. Professor Yang Hongtao (Fu, 2021), when treating patients with chronic kidney disease stages III–IV, used EA as a core drug to reduce serum creatinine and blood urea nitrogen. Chang et al. (2012) found that in a unilateral nephrectomy combined with STZ-induced DN rat model, EA alleviated glomerulosclerosis and renal function injury by downregulating transforming growth factor-*β*1 expression, and reducing the deposition of extracellular matrix metabolites.

Diabetic retinopathy Treatment: Li et al. (2021) used the Blood-Activating and Detoxifying Formula (with EA as one of the main metabolites) to improve diabetic retinopathy in STZ-induced DM rats. The study found it increased peak systolic velocity and end-diastolic velocity of the retina, reduced resistance index, and increased retinal thickness and number of ganglion cells while reducing apoptosis. Further mechanistic studies revealed that diabetes induced upregulation of 11 microRNAs and downregulation of four microRNAs in the rat retina, and Huoxue Jiedu Formula containing EA significantly reversed these dysregulated miRNAs, including the key molecule microRNA-423–5p. Combined with findings from (Xiao et al., 2019), treatment with the Blood-Activating and Detoxifying Formula reversed these changes: microRNA-423–5p reduced high-glucose-induced apoptosis of retinal pigment epithelial cells by inhibiting the trefoil factor family 1-mediated NF-*κ*B signaling pathway (Li et al., 2021). Wang et al. (2022) found that the water extract of EA delayed diabetic retinopathy progression by downregulating seven angiogenesis-related genes (e.g., matrix metalloproteinase 2, a disintegrin and metalloproteinase with thrombospondin motifs 15), and thereby suppressing pathological angiogenesis.

**FIGURE 6:**
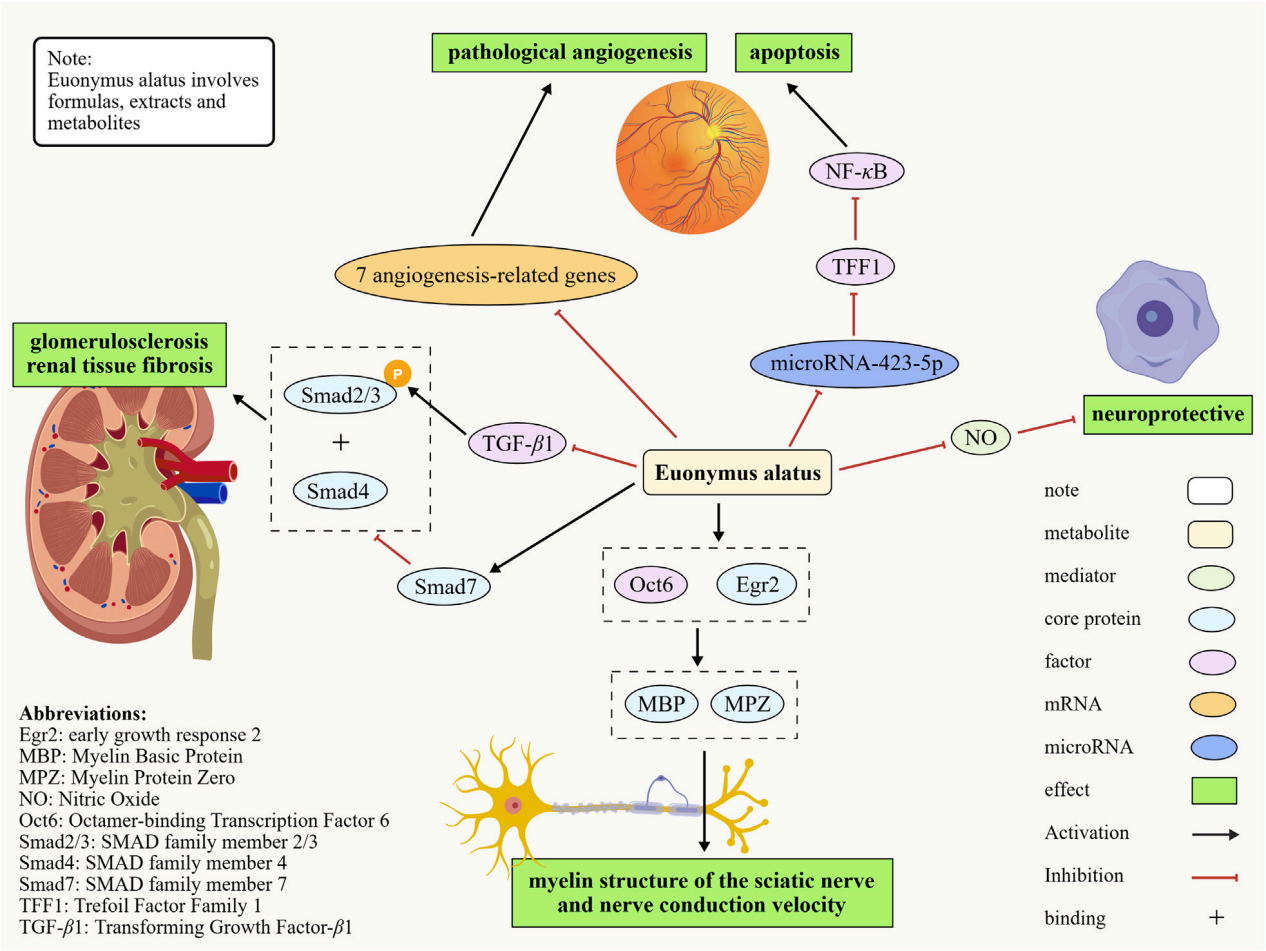
Mechanism of EA against diabetic complications.

DM-Related Neurological Issues: Hao et al. (2017) found that active plant metabolites of EA, *Trichosanthes kirilowii* Maxim. (Cucurbitaceae; official pharmacopoeial name: Trichosanthis Radix (Tianhuafen), *Panax notoginseng* (Burkill) F.H.Chen (Araliaceae; official pharmacopoeial name: Notoginseng Radix et Rhizoma (Sanqi) and *C. chinensis* improved diabetic peripheral neuropathy: these plant metabolites promoted synthesis of myelin basic protein and myelin protein zero by upregulating octamer-binding transcription factor 6 and early growth response protein 2, thereby improving myelin structure of the sciatic nerve and nerve conduction velocity. Jeong et al. (2011a) found that the 80% methanol extract of EA significantly inhibited LPS-induced nitric oxide generation in BV2 microglia. Notably, flavonoids (e.g., kaempferol, quercetin) showed particularly strong inhibitory activity, even superior to the positive control, indicating EA’s potential efficacy in treating DM encephalopathy. Furthermore, Gurung et al. (2024) reported that EA exhibited neuroprotective potential. You et al. (2024) found that EA inhibited microtubule-associated protein Tau hyperphosphorylation, reduced neurofibrillary tangle formation, alleviated neuronal cytoskeletal damage, and thereby improving cognitive decline. These findings point to the need for further mechanistic studies of central nervous system complications associated with DM. Mechanism of EA against diabetic complications is illustrated in the Figure 6 below.

## 6 Conclusion and perspectives

EA is one of the commonly used Chinese herbal medicines for the treatment of diabetes in clinical practice. Its hypoglycemic, lipid-regulating, and target-organ protective effects have been relatively extensively studied in diabetic animal models. To date, more than 230 metabolites, including flavonoids, terpenoids, phenylpropanoids, lignans, and alkaloids, have been isolated and identified from this botanical drug. Among them, quercetin, kaempferol, rutin, protocatechuic acid, caffeic acid, chlorogenic acid, catechin, ellagic acid, *β*-sitosterol, luteolin, apigenin, linarin, betulin, and catechu lactone A, among others, have been demonstrated to possess anti-diabetic activities. The underlying mechanisms involve: inhibition of intestinal *α*-glucosidase activity, delaying intestinal glucose absorption; activation of the PPAR*γ* and PI3K/AKT signaling pathways, promoting glucose transporter four translocation and improving insulin resistance; modulation of gut microbiota composition (reducing the Firmicutes/Bacteroidetes ratio and increasing the abundances of *Lactobacillus* and Akkermansia), thereby increasing short-chain fatty acid production; suppression of the AGEs-RAGE axis and the NF-*κ*B and mitogen-activated protein kinase inflammatory pathways, alleviating oxidative stress and inflammation; enhancement of antioxidant enzyme expression through the nuclear factor erythroid 2-related factor 2 and antioxidant response element pathway, protecting pancreatic *β* cells and renal tubular epithelial cells; regulation of DGAT activity, reducing lipid deposition; attenuation of glomerulosclerosis through the transforming growth factor-*β*1-SMAD family protein pathway; delay of diabetic retinopathy progression via inhibition of the PI3K/AKT pathway; and improvement of diabetic peripheral neuropathy by upregulating octamer-binding transcription factor 6 and early growth response protein 2 to promote myelin repair.

Despite the abundant preclinical findings, the current research system of EA still has prominent methodological limitations that hinder its clinical translation. In terms of phytochemical research, there is a lack of unified standards for medicinal material origin, harvesting, processing and extraction procedures across different studies. Most research focuses excessively on flavonoids, while the activity, structure-activity relationship and component interactions of triterpenoids, alkaloids and other constituents remain poorly explored, resulting in fragmented chemical evidence. For preclinical animal experiments, most models adopt single STZ-induced diabetic rodents with short intervention cycles, which cannot recapitulate the complex pathological features of long-term diabetes combined with multiple complications in clinical patients. In addition, most studies lack standardized dose-gradient tests for ED50 and LD50, making it difficult to establish reliable dose-effect relationships for human medication. Existing experiments only detected changes in downstream phenotypic indicators and failed to clarify the causal relationship between glucose metabolism, inflammation and oxidative stress via gene knockout or pathway blocking approaches, leading to superficial mechanistic interpretations. Systematic reports on classical pharmacokinetic parameters are lacking. Targeted pharmacokinetic studies are therefore warranted, including ultra-performance liquid chromatography–tandem mass spectrometry-based quantification of quercetin, kaempferol, and rutin in plasma and tissues; comparative activity evaluation and target engagement validation using orthogonal assay methods for representative metabolites with diverse skeletons; systematic isolation and pharmacological screening of triterpenoids, lignans, phenylpropanoids, and other non-flavonoid metabolites; determination of absolute bioavailability; identification of phase I/II metabolites; and characterization of gut microbiota-mediated biotransformation. Such systematic investigations are essential to clarify the *in vivo* fate and active substances of EA metabolites. Furthermore, the observed anti-diabetic effects may be attributed not only to parent metabolites but also to their phase I/II metabolites or gut microbiota-mediated biotransformation products, which requires further identification and validation. Such experimental design defects mean current preclinical evidence can only support preliminary activity screening, rather than drawing definitive conclusions on therapeutic efficacy for chronic diabetic complications.

Clinical research also faces obvious deficiencies. The Grading of Recommendations Assessment, Development and Evaluation was used to assess the certainty of clinical evidence in this study. The overall quality of evidence was judged as low, mainly because of high inter-study heterogeneity, limited sample size and potential methodological bias of original literature. Potential publication bias cannot be excluded because most available published studies reported positive outcomes. Noticeable clinical and methodological heterogeneity exists among the included studies, due to differences in age, disease course, and baseline conditions of participants, diverse medication dosage and treatment duration, as well as inconsistent outcome evaluation criteria. These discrepancies reduce the reproducibility of the pooled results and imply that the clinical effect of EA may be population-specific. Small sample sizes, non-rigorous trial design, inconsistent evaluation criteria and short follow-up periods result in low-quality clinical evidence with high heterogeneity. Moreover, long-term safety assessment of EA is insufficient. Systemic investigations on hepatotoxicity, nephrotoxicity and adverse reactions in vulnerable populations such as elderly patients and patients with liver and kidney dysfunction are still absent. Although EA shows no obvious hypoglycemia and weight gain side effects compared with conventional anti-diabetic drugs, head-to-head clinical trials are still required to verify its risk-benefit profile. Moreover, almost all clinical observations are based on multi-herb compound formulas rather than single EA preparations. These limitations do not imply that single-agent evaluation can be omitted; on the contrary, rigorous monotherapy studies must be prioritized in future research to comply with standard pharmacological principles. For this reason, all conclusions should be interpreted cautiously, and definitive conclusions regarding its efficacy cannot yet be drawn.

All findings presented in this review are based on preclinical and limited clinical evidence, and none of the conclusions support immediate clinical application. To this end, future research urgently requires: first, systematic *in vivo* pharmacokinetic investigations to elucidate the absorption, distribution, metabolism, and excretion profiles of the principal bioactive metabolites, with consideration of gut microbiota heterogeneity and interindividual variability; second, the application of emerging technologies, including metabolomics, gut microbiota metagenomics, and epigenetics, to further delineate novel anti-diabetic mechanisms of EA and clarify the impacts of genetic polymorphisms and microbiome differences on therapeutic outcomes; third, rigorously designed multi-center, large-scale randomized controlled trials to evaluate the therapeutic efficacy and safety of EA as a monotherapy or its standardized extracts in diabetic populations, which should be prospective, double-blind, placebo-controlled, multi-center RCT enrolling patients with type 2 diabetes mellitus. Primary endpoints should include changes in fasting blood glucose, glycated hemoglobin, and postprandial blood glucose; secondary endpoints should encompass insulin resistance indices, lipid profiles, inflammatory markers, and incidence of adverse events. The treatment duration should be 12–24 weeks, with a run-in period of 2–4 weeks. The control arms should include a placebo group and an active comparator group receiving established first-line anti-diabetic therapy (e.g., metformin), to enable assessment of both efficacy relative to placebo and non-inferiority compared to standard care. Additionally, dose-response evaluation across at least three dosage levels would be essential to establish the optimal therapeutic dose range; and fourth, intensified structure-activity relationship studies to define how distinct chemical architectures contribute to anti-diabetic activity, thereby informing rational drug design. These directions fully align with the four pillars of best practice in ethnopharmacology, emphasizing authentic plant identification, standardized experimental protocols, transparent scientific reporting, and mechanism-focused evidence validation. The advancement of these research endeavors will provide critical guidance for the optimal clinical utilization of EA in mitigating the escalating challenge of diabetes treatment failure.

## References

[B1] Acharya B. Kaur P. Srivastava D. Chaudhary P. Sharma N. Arya V. (2024). Unveiling the medicinal potential of *Euonymus alatus* (thunb.) siebold: from traditional knowledge to mechanistic understanding. Pharmacol. Res. Nat. Prod. 5, 100102. 10.1016/j.prenap.2024.100102

[B2] Acosta-Martinez M. Cabail M. Z. (2022). The PI3K/Akt pathway in meta-inflammation. Int. J. Mol. Sci. 23, 15330. 10.3390/ijms232315330 36499659 PMC9740745

[B3] Ba Y. Y. Shi R. B. Liu Q. Y. Zhang L. Z. (2012). Chemical compositions of guijianyu (*ramulus euonymi alatae*). J. Beijing Univ. Tradit. Chin. Med. 35, 480–483.

[B4] Bansal S. Durrett T. P. (2016). Defining the extreme substrate specificity of *Euonymus alatus* diacylglycerol acetyltransferase, an unusual membrane-bound O-acyltransferase. Biosci. Rep. 36, e00406. 10.1042/BSR20160277 27688773 PMC5100001

[B5] Bolz S. N. Adasme M. F. Schroeder M. (2021). Toward an understanding of pan-assay interference compounds and promiscuity: a structural perspective on binding modes. J. Chem. Inf. Model 61, 2248–2262. 10.1021/acs.jcim.0c01227 33899463

[B6] Bu X. H. An H. Y. An X. N. Zhang C. C. Guo X. Y. (2021). Mechanism of *ramulus euonymi* in treatment of diabetic nephropathy based on network pharmacology and molecular docking. J. Hunan Univ. Chin. Med. 41, 1564–1573. 10.3969/j.issn.16740-70X.2021.10.017

[B7] Cha B.-Y. Park C.-J. Lee D.-G. Lee Y.-C. Kim D.-W. Kim J.-D. (2003). Inhibitory effect of methanol extract of *Euonymus alatus* on matrix metalloproteinase-9. J. Ethnopharmacol. 85, 163–167. 10.1016/s0378-8741(02)00373-2 12576216

[B8] Chang B. Jin C. Zhang W. Kong L. Yang J. H. Lian F. M. (2012). *Euonymus alatus* in the treatment of diabetic nephropathy in rats. Am. J. Chin. Med. 40 (6), 1177–1187. 10.1142/S0192415X12500875 23227790

[B9] Chen Y. H. Gong M. X. Zhang C. Song Y. F. Yu P. (2010). Determination of aromadendrin in *Euonymus alatus* by HPLC. China J. Chin. Mater Med. 35, 2565–2567. 10.4268/cjcmm20101914 21174766

[B10] Chen J. H. Sun J. Jin L. P. Gu L. R. Dai D. Z. (2018). Observation on the hypoglycemic effect of zingerone and its composition in diabetic mice. Jiangsu Agric. Sci. 46, 166–168. 10.15889/j.issn.1002-1302.2018.10.042

[B11] Chen M. H. Wang Y. L. Li X. G. Lyu H. F. Huang D. B. (2022). Ethanolic extracts of *Euonymus alatus* reduce renal ischemia-reperfusion injury in rabbits by preventing oxidative stress and inhibiting TNF-*α*-NF-*κ*B and T*β*R1-Smad2/3 signaling pathways. Chin. J. Pathophysiol. 38, 688–697. 10.3969/j.issn.1000-4718.2022.04.015

[B12] Chung H.-S. Jeong H.-J. Kim J.-S. Jeong S.-I. Kim K.-S. Kim K.-S. (2002). Activation of inducible nitric oxide synthase by *Euonymus alatus* in mouse peritoneal macrophages. Clin. Chim. Acta 318, 113–120. 10.1016/s0009-8981(01)00808-7 11880120

[B13] Dang X. F. Cao S. L. Qi J. J. Zhang H. Zhang J. Fu J. (2014). Optimization of determination for total flavonoids in *Euonymus alatus* . Chin. J. Exp. Tradit. Med. Formulae 20, 96–98. 10.13422/j.cnki.syfjx.2014090096

[B14] Duan Y. H. Wu M. (2014). Experimental study of the effect of *Euonymus alatus* compatibility litchi on glucolipide metabolism of drosophila model. Jilin J. Tradit. Chin. Med. 34, 278–281. 10.13463/j.cnki.jlzyy.2014.03.021

[B15] Durrett T. P. McClosky D. D. Tumaney A. W. Elzinga D. A. Ohlrogge J. Pollard M. (2010). A distinct DGAT with *sn*-3 acetyltransferase activity that synthesizes unusual, reduced-viscosity oils in euonymus and transgenic seeds. Proc. Natl. Acad. Sci. 107 (20), 9464–9469. 10.1073/pnas.1001707107 20439724 PMC2889089

[B16] Fan L. H. Zhang C. L. Ai L. Wang L. Li L. Fan W. X. (2020). Traditional uses, botany, phytochemistry, pharmacology, separation and analysis technologies of *Euonymus alatus* (thunb.) siebold: a comprehensive review. J. Ethnopharmacol. 259, 112942. 10.1016/j.jep.2020.112942 32423879

[B17] Fang Z. F. (2007). “Studies on the chemical constituents and antitumor activities of *Euonymus alatus* ,”. Shenyang (LN): Shenyang Pharmaceutical University.

[B18] Fang X. K. Gao J. Zhu D. N. (2008). Kaempferol and quercetin isolated from *Euonymus alatus* improve glucose uptake of 3T3-L1 cells without adipogenesis activity. Life Sci. 82, 615–622. 10.1016/j.lfs.2007.12.021 18262572

[B19] Fu B. H. (2021). “Data mining and network pharmacological analysis of the prescriptions by professor yang hongtao for the treating CKD stage 3–4,”. Tianjin (TJ): Tianjin University of Traditional Chinese Medicine. 10.27368/d.cnki.gtzyy.2021.000388

[B20] Gieroba B. Kryska A. Sroka-Bartnicka A. (2025). Type 2 diabetes Mellitus—Conventional therapies and future perspectives in innovative treatment. Biochem. Biophys. Rep. 42, 102037. 10.1016/j.bbrep.2025.102037 40395625 PMC12090304

[B21] Gurung P. Shrestha R. Lim J. Thapa Magar T. B. Kim H.-H. Kim Y.-W. (2023). *Euonymus alatus* twig extract protects against scopolamine-induced changes in brain and brain-derived cells via cholinergic and BDNF pathways. Nutrients 15, 128. 10.3390/nu15010128 PMC9823662 36615789

[B22] Gurung P. Lim J. Thapa Magar T. B. Shrestha R. Kim Y.-W. (2024). *Euonymus alatus* leaf extract attenuates effects of aging on oxidative stress, neuroinflammation, and cognitive impairment. Antioxidants 13, 433. 10.3390/antiox13040433 38671881 PMC11047375

[B23] Hao G. M. Liu Y. G. Wu Y. Xing W. Guo S. Z. Wang Y. (2017). The protective effect of the active components of ERPC on diabetic peripheral neuropathy in rats. J. Ethnopharmacol. 202, 162–171. 10.1016/j.jep.2017.03.015 28315720

[B24] He S.-Y. Qiu X.-M. Wang Y.-Q. Su Z.-Q. Zhang B.-Y. Wen Z. (2022). Intervention effect of *potentilla discolor-Euonymus alatus* on intestinal flora of type 2 diabetes mellitus rats. Eur. Rev. Med. Pharmacol. Sci. 26, 9062–9071. 10.26355/eurrev_202212_30655 36591818

[B25] Huang L. Xiong W. H. Wang H. Q. Zou F. Y. Tang Z. H. Zhang C. (2014). Aqueous components of *Euonymus alatus* inhibit the expression of inflammatory factors in THP-1-derived macrophages induced by oxidized low-density lipoprotein. Chin. J. Mod. Drug Appl. 8, 250–251. 10.14164/j.cnki.cn11-5581/r.2014.14.018

[B26] International Diabetes Federation (2025). IDF Diabetes Atlas. 11th edn. Brussels, Belgium: International Diabetes Federation. Available online at: https://idf.org/news/idf-diabetes-atlas-11th-edition/ (Accessed March 29, 2026).

[B27] Jeong E. J. Yang H. Kim S. H. Kang S. Y. Sung S. H. Kim Y. C. (2011a). Inhibitory constituents of *Euonymus alatus* leaves and twigs on nitric oxide production in BV2 microglia cells. Food Chem. Toxicol. 49, 1394–1398. 10.1016/j.fct.2011.03.028 21426922

[B28] Jeong E. J. Cho J. H. Sung S. H. Kim S. Y. Kim Y. C. (2011b). Inhibition of nitric oxide production in lipopolysaccharide-stimulated RAW264.7 macrophage cells by lignans isolated from *Euonymus alatus* leaves and twigs. Bioorg Med. Chem. Lett. 21, 2283–2286. 10.1016/j.bmcl.2011.02.102 21435874

[B29] Jeong S.-Y. Nguyen P.-H. Zhao B.-T. Ali M. Y. Choi J.-S. Min B.-S. (2015). Chemical constituents of *Euonymus alatus* (thunb.) sieb. and their PTP1B and *α*-Glucosidase inhibitory activities. Phytother. Res. 29, 1540–1548. 10.1002/ptr.5411 26172104

[B30] Ji R. F. Du Y. Wang Y. Tian J. Y. Wang Z. L. Peng M. Z. (2024). Stigmast-4-en-3-one from *Euonymus alatus* (thunb.) siebold. Improves diabetic retinopathy and angiogenesis mediated by glucocorticoids receptor. J. Ethnopharmacol. 334, 118567. 10.1016/j.jep.2024.118567 38996951

[B31] Jin U. H. Lee J. Y. Kang S. K. Kim J. K. Park W. H. Kim J. G. (2005). A phenolic compound, 5-caffeoylquinic acid (chlorogenic acid), is a new type and strong matrix metalloproteinase-9 inhibitor: isolation and identification from methanol extract of *Euonymus alatus* . Life Sci. 77, 2760–2769. 10.1016/j.lfs.2005.02.028 16005473

[B32] Jung Y. S. Lee H. W. Ma C. J. (2020). Neuroprotective effect of compounds isolated from *Euonymus alatus* on glutamate-induced oxidative stress in HT22 hippocampal cells. Phcog Mag. 16, S308–S314. 10.4103/pm.pm_450_19

[B33] Kim Y. Cho M. Lee J. S. Oh J. Lim J. (2024). Protocatechuic acid from *Euonymus alatus* mitigates scopolamine-induced memory impairment in mice. Foods 13, 2664. 10.3390/foods13172664 39272430 PMC11394611

[B34] Lee S.-K. Hwang J.-Y. Song J.-H. Jo J.-R. Kim M.-J. Kim M.-E. (2007). Inhibitory activity of *Euonymus alatus* against alpha-glucosidase *in vitro* and *in vivo* . Nutr. Res. Pract. 1 (3), 184–188. 10.4162/nrp.2007.1.3.184 20368936 PMC2849020

[B35] Lee S. Lee D. Baek S. C. Jo M. S. Kang K. S. Kim K. H. (2019). (3*β*,16*α*)-3,16-Dihydroxypregn-5-en-20-one from the twigs of *Euonymus alatus* (thunb.) sieb. Exerts anti-inflammatory effects in LPS-stimulated RAW-264.7 macrophages. Molecules 24, 3848. 10.3390/molecules24213848 31731472 PMC6864714

[B36] Lee S.-U. Gurung P. Thapa Magar T. B. Lim J. Shrestha R. Kim Y.-H. (2024). *Euonymus alatus* extract reduces insulin resistance in Db/Db mice by regulating the PI3K-AKT pathway. Int. J. Transl. Med. 4, 286–297. 10.3390/ijtm4020018

[B37] Li X. X. (2024). “Case study of professor zhou zhongying, a master of national medicine, based on data mining in the treatment of diabetes mellitus,”. Nanjing (JS): Nanjing University of Chinese Medicine. 10.27253/d.cnki.gnjzu.2024.000529

[B38] Li J. E. Wang L. Qin L. L. Liu T. H. (2010). Effects of winged *euonymus* twig on insulin resistance and adipo-cytokines in T2DM rats. Guid J. Tradit. Chin. Med. Pharm. 16, 1–3. 10.13862/j.cnki.cn43-1446/r.2010.11.033

[B39] Li Y. J. Gong M. X. Lai Y. Y. Wang Q. Chen Y. H. Tian P. F. (2010). Pharmacological effects of different extract fractions from guijianyu (*Euonymus alatus*) on diabetic rats. J. Beijing Univ. Tradit. Chin. Med. 33, 179–182.

[B40] Li H. L. Hao G. M. Tang S. J. Sun H. H. Fang Y. S. Pang X. X. (2021). HuoXue JieDu formula improves diabetic retinopathy in rats by regulating microRNAs. J. Ethnopharmacol. 268, 113616. 10.1016/j.jep.2020.13616 33271246

[B41] Li B. W. Huang J. K. Zhao Q. Y. Li J. Y. Zhang Y. Hu C. Y. (2024). Clinical experience *astragalus* and *Gymnema sylvestre* in the treatment of diaopathy. China Med. 19, 257–260. 10.3760/j.issn.1673-4777

[B42] Li Y. P. Zhang K. Wang Z. G. Yang X. Li J. J. Yu D. D. (2026). Research progress on the mechanism of *Euonymus alatus* and its active components in the treatment of diabetic kidney disease. J. Hainan Med. Univ. 32, 310–320. 10.13210/j.cnki.jhmu.20250708.003

[B43] Liang P. M. Zhang G. X. (2001). Summary of 57 cases of dawn phenomenon in type II diabetes mellitus treated with modified leishi aromatic resolving turbidity formula. Hunan J. Tradit. Chin. Med. 17, 16–17. 10.16808/j.cnki.issn1003-7705.2001.02.014

[B44] Liu H. Y. Leng W. (2018). Clinical observation of qiyu tangmaikang in the treatment of stage III–IV dia angming J. Chin. Med. 33, 484–486. 10.3969/j.issn.1003-8914.2018.04.016

[B45] Liu C. J. Yang J. X. Fu W. J. Qi S. Q. Wang C. M. Quan C. (2014). Coactivation of the PI3K/Akt and ERK signaling pathways in PCB153-induced NF-*κ*B activation and caspase inhibition. Toxicol. Appl. Pharmacol. 277, 270–278. 10.1016/j.taap.2014.03.027 24726520

[B46] Ma Q. Q. Niu Y. Q. Zuo M. Y. Li X. Fu J. K. Wang J. J. (2025). Guijianyu alleviates advanced glycation endproducts-induced mouse renal podocyte injury by inhibiting the AGEs-RAGE South Med. Univ. 45 (09.13), 1938–1945. 10.12122/j.issn.1673-4254.2025 PMC12479273 41022603

[B47] Mai X. M. Liu Y. T. Tang X. Y. Wang L. P. Lin Y. Y. Zeng H. Y. (2020). Sequential extraction and enrichment of flavonoids from *Euonymus alatus* by ultrasonic-assisted polyethylene glycol-based extraction coupled to temperature-induced cloud point extraction. Ultrason. Sonochem 66, 105073. 10.1016/j.ultsonch.2020.105073 32247232

[B48] Milcamps A. Tumaney A. W. Paddock T. Pan D. A. Ohlrogge J. Pollard M. (2005). Isolation of a gene encoding a 1,2-diacylglycerol-sn-acetyl-CoA acetyltransferase from developing seeds of *Euonymus alatus* . J. Biol. Chem. 280 (7), 5370–5377. 10.1074/jbc.M410276200 15579902

[B49] NCD Risk Factor Collaboration (NCD-RisC) (2024). Worldwide trends in diabetes prevalence and treatment from 1990 to 2022: a pooled analysis of 1108 population-representative studies with 141 million participants. Lancet 404, 2077–2093. 10.1016/S0140-6736(24)02317-1 39549716 PMC7616842

[B50] Oh B. K. Mun J. Seo H. W. Ryu S. Y. Kim Y. S. Lee B. H. (2011). *Euonymus alatus* extract attenuates LPS-Induced NF-*κ*B activation *via* IKK*β* inhibition in RAW 264.7 cells. J. Ethnopharmacol. 134, 288–293. 10.1016/j.jep.2010.12.020 21182917

[B51] Park W. H. Kim S. H. Kim C. H. (2005). A new matrix metalloproteinase-9 inhibitor 3,4-dihydroxycinnamic acid (caffeic acid) from methanol extract of *euonymus Alatus*: isolation and structure determination. Toxicology 207, 383–390. 10.1016/j.tox.2004.10.008 15664266

[B52] Park S. H. Ko S. K. Chung S. H. (2005). *Euonymus alatus* prevents the hyperglycemia and hyperlipidemia induced by high-fat diet in ICR mice. J. Ethnopharmacol. 102, 326–335. 10.1016/j.jep.2005.06.041 16095853

[B53] Qi F. Liu G. S. She J. Ji X. M. Zheng H. Z. Gong M. X. (1998). Effects of *Euonymus alatus* on blood glucose and blood lipid in experimental diabetic mice. Chin. J. Inf. Tradit. Chin. Med. 5, 19–20.

[B54] Qi Z. L. Bai M. W. Bai P. Yang S. Y. (2000). Clinical observation of compound *Euonymus alatus* mixture in the treatment of chronic nephritis. Henan J. Prev. Med. 11, 64. 10.13515/j.cnki.hnjpm.2000.01.045

[B55] Seo U.-K. Lee Y.-J. Kim J.-K. Cha B.-Y. Kim D.-W. Nam K.-S. (2005). Large-scale and effective screening of Korean medicinal plants for inhibitory activity on matrix metalloproteinase-9. J. Ethnopharmacol. 97, 101–106. 10.1016/j.jep.2004.10.022 15652283

[B56] Shin D. Y. Kim B. S. Lee H. Y. Park Y. M. Kim Y. W. Kim M. J. (2023). *Euonymus alatus* (thunb.) siebold leaf extract enhanced immunostimulatory effects in a cyclophosphamide-induced immunosuppressed rat model. Food Nutr. Res. 67, 9422. 10.29219/fnr.v67.9422 37152296 PMC10155189

[B57] Song C. (2020). “The experience of guo junjie in the treatment of prediabetes and patterns of Chinese herbs,”. Jinzhong (SX): Shanxi University of Chinese Medicine. 10.27820/d.cnki.gszxy.2020.000145

[B58] Su K. L. (2011). “The retrospective research on the cases of professor zhou zhongying treating DN based on data mining,”. Nanjing (JS): Nanjing University of Chinese Medicine.

[B59] Sun N. Wang Y. J. Zhou C. H. Kang H. H. Wang R. Feng X. (2025). A review of pharmacological effects and bioactive constituents of *Euonymus alatus* . Phytochem. Lett. 68, 103003. 10.1016/j.phytol.2025.103003

[B60] Tian T. L. Wang S. S. Chen X. Y. (2014). Experience of professor ZHOU Zhong-ying in treating diabetes. Clin. J. Chin. Med. 6, 113–114. 10.3969/j.issn.1674-7860.2014.07.062

[B61] Wan X. Huang H. C. Wang X. P. Hu Z. H. Liu K. Y. Huang D. B. (2019). *Euonymus alatus* and its monomers alleviate liver fibrosis both in mice and LX2 cells by blocking T*β*R1-Smad2/3 and TNF-*α*-NF-*κ*B pathways. Am. J. Transl. Res. 11 (1), 106–119. 30787972 PMC6357326

[B62] Wang G. Q. (2022). “Protective effect of *Euonymus alatus* on vascular endothelial injury in spontaneously hypertensive rats by interfering with PERK signaling pathway,”. Changsha (HN): Hunan University of Chinese Medicine. 10.27138/d.cnki.ghuzc.2022.000106

[B63] Wang W. J. Liu Y. Y. Che F. F. Li H. Liu J. G. Wu N. (2021). Isolation and purification of flavonoids from *Euonymus alatus* by high-speed countercurrent chromatography and neuroprotective effect of rhamnazin-3-O-rutinoside *in vitro* . J. Sep. Sci. 44, 4422–4430. 10.1002/jssc.202100607 34670011

[B64] Wang Z. L. Sun H. H. Liu H. Y. Ji Q. X. Niu Y. T. Ma P. (2022). The water extracts of *Euonymus alatus* (thunb.) siebold attenuate diabetic retinopathy by mediating angiogenesis. J. Ethnopharmacol. 284, 114782. 10.1016/j.jep.2021.114782 34728316

[B65] Wang J. J. Cui W. F. Dou X. W. Yin B. L. Niu Y. Q. Niu L. (2024). *Euonymus alatus* delays progression of diabetic kidney disease in mice by regulating EGFR tyrosine kinase inhibitor resistance signaling pathway. J. South Med. Univ. 44 (7), 1243–1255. 10.12122/j.issn.1673-4254.2024.07.04 PMC11270662 39051070

[B66] Wang J. J. Niu Y. Q. Ma Q. Q. Zuo M. Y. Yan G. L. (2025). Study on mechanism of *Euonymus alatus* intervention in diabetic kidney disease mice based on AGEs-RAGE signal transduction pathway. Chin. J. Prev. Contr Chron. Dis. 33, 126–134. 10.16386/j.cjpccd.issn.1004-6194.20240710.0498

[B67] Woo Y. Lim J. S. Oh J. Lee J. S. Kim J.-S. (2020). Neuroprotective effects of *Euonymus alatus* extract on scopolamine-induced memory deficits in mice. Antioxidants 9, 449. 10.3390/antiox9050449 32456069 PMC7278771

[B68] Wu Q. J. (2021). “Exploring the effect and mechanism of qingli huoxue method in treating chronic kidney disease based on “adriamycin nephropathy” model,” in Dissertation. Nanjing (JS): Nanjing University of Chinese Medicine. 10.27253/d.cnki.gnjzu.2021.000069

[B69] Xiao G. D. (2013). “Studies on chemical constituents of *Astragalus membranaceus* and *Euonymus alatus* ,”. Hangzhou (ZJ): Zhejiang University.

[B70] Xiao Q. Zhao Y. Xu J. Li W. J. Chen Y. Sun H. J. (2019). NFE2/miR-423-5p/TFF1 axis regulates high glucose-induced apoptosis in retinal pigment epithelial cells. BMC Mol. Cell Biol. 20, 39. 10.1186/s12860-019-0223-2 31455213 PMC6712806

[B71] Xing S. L. Huang W. Z. Zheng J. Y. (2005). Clinical and experimental research progress of *Euonymus alatus* . Jilin J. Tradit. Chin. Med. 25, 62–63. 10.13463/j.cnki.jlzyy.2005.11.048

[B72] Yamashita H. Matsuzaki M. Kurokawa Y. Nakane T. Goto M. Lee K.-H. (2019). Four new triterpenoids from the bark of *Euonymus alatus* forma *Ciliato-dentatus* . Phytochem. Lett. 31, 140–146. 10.1016/j.phytol.2019.03.015 31379978 PMC6677265

[B73] Yang Y. Zhang F. (2008). Ultrasound-assisted extraction of rutin and quercetin from *Euonymus alatus* (thunb.) sieb. Ultrason. Sonochem 15, 308–313. 10.1016/j.ultsonch.2007.05.001 17606398

[B74] Yang H. Y. Wang Q. J. Zhu D. N. Zhao X. C. (2004). Effect of extracts from *Euonymus alatus* sieb. On peripheral glucose uptake of adipocytes. Chin. J. Nat. Med. 2, 49–52.

[B75] Yoon W.-J. Min H.-J. Cho H.-D. Kim H.-G. Park W.-L. Kim D.-H. (2024). Bioactivity evaluation and phytochemical characterization of *Euonymus alatus* (thunb.) siebold leaf extract. Biomedicines 12, 2928. 10.3390/biomedicines12122928 39767833 PMC11673389

[B76] You Y.-L. Byun H.-J. Lee J. S. Choi H.-S. Youk J.-S. (2024). *Euonymus alatus* and its compounds suppress hydrogen peroxide-induced oxidative stress in HT22 cells. Food Sci. Biotechnol. 33, 3567–3577. 10.1007/s10068-024-01601-4 39493395 PMC11525359

[B77] Zhai X. F. Lenon G. B. Xue C. C. L. Li C. G. (2016). *Euonymus Alatus*: a review on its phytochemistry and antidiabetic activity. Evid. Based Complement. Altern. Med. 2016, 9425714. 10.1155/2016/9425714 27642361 PMC5014951

[B78] Zhang F. (2007). “Ultrasound-assisted extraction and microwave-assisted extraction of flavones from *Euonymus alatus* sieb,”. Beijing (BJ): Beijing University of Chemical Technology.

[B79] Zhang Y. X. (2018). Overview of experimental research and clinical application of *Euonymus alatus* in diabetes mellitus. Gen. J. Stomatol. 5, 31–32. 10.16269/j.cnki.cn11-9337/r.2018.32.015

[B80] Zhang F. Yang Y. Su P. Guo Z. K. (2009). Microwave-assisted extraction of rutin and quercetin from the stalks of *Euonymus alatus* (thunb.) sieb. Phytochem. Anal. 20, 33–37. 10.1002/pca.1088 19086093

[B81] Zhang W. Zhen Z. Huang W. Z. Zhu X. D. (2011). Effect of *euonymi ramuli* compound on expression of Smad4 and Smad7 in nephridial tissue of rat with diabetic nephropathy. Shenzhen J. Integr. Tradit. Chin. West Med. 21, 129–132+193. 10.16458/j.cnki.1007-0893.2011.03.010

[B82] Zhang L. J. Zhu J. Y. Sun M. Y. Song Y. N. Rahman K. Peng C. (2017). Anti-inflammatory effect of man-pen-fang, a Chinese herbal compound, on chronic pelvic inflammation in rats. J. Ethnopharmacol. 208, 57–65. 10.1016/j.jep.2017.06.034 28652014

[B83] Zhao M. M. Xie M. Z. Li L. D. He J. F. Wang Y. (2010). Effects of *Euonymus alatus* on islet *β* cell in type 2 diabetic rats. J. Hunan Univ. Chin. Med. 30, 14–16.

[B84] Zhou Y. M. Xia M. Yin T. T. Zhang Y. N. Huang Q. R. (2021). Aromadendrin improves insulin resistance in vascular endothelial cells via PPAR*γ* mediation. Jiangxi Med. J. 56, 269–272. 10.3969/j.issn.1006-2238.2021.03.001

[B85] Zhu Y. J. (1999). Clinical observation of 100 cases of diabetes II treated from the theory of liver. Shanghai J. Tradit. Chin. Med. 7, 19–20. 10.16305/j.1007-1334.1999.07.008

